# A randomised clinical trial study assessing the efficacy of 5% losartan potassium loaded in ethosomal gel to treat human keloids: a trial protocol

**DOI:** 10.1186/s13063-023-07880-2

**Published:** 2024-01-02

**Authors:** Yuni Eka Anggraini, Niken Trisnowati, Ronny Martien, Retno Danarti

**Affiliations:** 1 Faculty of Medicine, Universitas Riau, Kota Pekanbaru, Indonesia; 2 Faculty of Medicine, Public Health, and Nursing, Universitas Gadjah Mada, Yogyakarta, Indonesia; 3 Faculty of Pharmacy, Universitas Gadjah Mada, Yogyakarta, Indonesia

**Keywords:** Angiotensin II, Angiotensin II receptor blocker, Losartan potassium, Triamcinolone acetonide, Efficacy, Randomised clinical trial

## Abstract

**Background:**

Keloid is a skin disorder that results from excessive fibrous tissue growth in the area of the initial trauma. Treating keloids can be challenging since the success of various treatments varies from one study to another. Triamcinolone acetonide injection, a standard treatment, can cause undesirable side effects. Meanwhile, the effectiveness of existing topical therapies for keloids is not always reliable. The pro-inflammatory, pro-proliferative, and pro-fibrotic effects of angiotensin II in human skin contribute to keloid formation. Losartan potassium, an angiotensin II blocker, has the potential to act as an anti-keloid agent. Due to the thicker skin structure of a keloid and ease of application, ethosome gel is chosen as a safe and comfortable carrier for losartan potassium, making it a good choice for treating keloids.

**Methods:**

In this randomised clinical trial, 46 adults with keloids were divided into two treatment groups. One group of 23 participants received 5% losartan potassium loaded in ethosomal gel, while the other group of 23 participants received intralesional injections of 10% triamcinolone acetonide. Over 12 weeks, changes in POSAS 3.0 scores, degree of erythema and pigmentation, surface area, thickness, and pliability of the keloids will be measured at four different times: baseline, 4 weeks, 8 weeks, and 12 weeks. Statistical analysis will be conducted using SPSS software version 24, with a significance level of *p* < 0.05.

**Discussion:**

Losartan potassium is believed to be beneficial for keloid management because it inhibits the angiotensin II receptor, which plays a role in inflammation, proliferation, and fibrosis. This study examines the efficacy of 5% losartan potassium loaded in ethosomal gel for human keloids.

**Trial registration:**

Clinicaltrial.gov identifier NCT05893108. Registered on 7 June 2023.

## Administrative information

Note: the numbers in curly brackets in this protocol refer to SPIRIT checklist item numbers. The order of the items has been modified to group similar items (see http://www.equator-network.org/reporting-guidelines/spirit-2013-statement-defining-standard-protocol-items-for-clinical-trials/).

**Table:** 

Title {1}	A randomised clinical trial study assessing the efficacy of 5% losartan potassium loaded in ethosomal gel to treat human keloids: a trial protocol.
Trial registration {2a and 2b}.	Clinicaltrial.gov: NCT05893108. https://clinicaltrials.gov/study/NCT05893108
Protocol version {3}	Issue Date 01 June 2023, Protocol amandement 00, Authors: YA, NT, RM, RD, CT202300, 2023-Jan-11 Original
Funding {4}	Final Project Recognition Grant Universitas Gadjah Mada Number 5075/UN1.P.II/DitLit/PT.01.01/2023.
Author details {5a}	(1) Yuni Eka Anggraini. MD, M.Med.Ed. Faculty of Medicine of Universitas Riau, Kota Pekanbaru, Indonesia; Faculty of Medicine, Public Health, and Nursing, Universitas Gadjah Mada Yogyakarta, Indonesia. (2) Niken Trisnowati. MD, M. Sc, Dr. Faculty of Medicine, Public Health, and Nursing, Universitas Gadjah Mada Yogyakarta, Indonesia. (3) Ronny Martien. Dr.rer.nat. Faculty of Pharmacy Universitas Gadjah Mada Yogyakarta, Indonesia. (4) Retno Danarti. MD, Dr Med., Prof. Faculty of Medicine, Public Health, and Nursing, Universitas Gadjah Mada Yogyakarta, Indonesia (corresponding author).
Name and contact information for the trial sponsor {5b}	Trial Sponsor: Sponsor-investigator Contact name: Yuni Eka Anggraini Address: Faculty of Medicine of Universitas Riau, Indonesia. Email: yuni.eka@lecturer.unri.ac.id
Role of sponsor {5c}	The funding source is not involved in the study’s design, execution, data analysis, or decision to submit results. The sponsor-investigator is responsible for designing the study, conducting it, analysing and interpreting the data, writing the manuscript, and disseminating the results.

## Introduction

### Background and rationale {6a}

Keloids and hypertrophic scars result from abnormal skin responses to trauma extending beyond the injury site and may appear years later [1]. Excessive scar tissue can cause pain, itching, and contractures that affect patients’ well-being [2, 3]. Keloid scarring is common among those who have had surgery or suffered trauma. Fifteen per cent of scar tissue can grow excessively, making treatment challenging. However, its prevalence and frequency data could be more extensive and scattered [4].

The cause of hypertrophic scars and keloids is not fully understood, but it is thought that one of the stages of wound healing is interrupted [5, 6]. Cytokines like interleukin (IL), e.g. IL-6, IL-8, and IL-10, may impact scar tissue formation. Furthermore, transforming growth factor-β (TGF-β) is not regulated well; collagen production can increase, leading to scarring. Imbalanced extracellular matrix deposition and degradation may also cause scars during remodelling [5, 7].

The renin-angiotensin system (RAS) is found in all layers of human skin [8], including cells like keratinocytes, fibroblasts, and vascular endothelial with angiotensin type I receptors (AT1R) and angiotensin type 2 receptors (AT2R). Only melanocytes have only AT1R receptors melanocytes [9–11]. Research showed that keloid fibroblasts have a higher expression of AT1R receptors than normal and hypertrophic scar skin [12]. Angiotensin is the ultimate player in the RAS process [13]. The liver produces angiotensinogen, which becomes angiotensin I with the help of renin. Angiotensin-converting enzyme (ACE) and chymase convert angiotensin I to angiotensin II, which has pro-inflammatory, pro-proliferation, and pro-fibrosis effects [14].

Treating keloid and hypertrophic scars is challenging, with no universally effective therapy available [15]. Intralesional triamcinolone acetonide (TAC) corticosteroid injections are the recommended therapy by the International Advisory Panel on Scar Management [16] and are widely used in the USA [17] and Indonesia. It is often used alone or with other modalities [17, 18] but may cause side effects like pain and skin changes [19]. Keloids often recur, even after excision [20]. Some people may be uncomfortable with needles [21]. Topical therapy for keloids and hypertrophic scars has limited options. Studies show varying results for topical drugs [22], but they are pain-free and easy to apply [23]. RAS-targeting topical medications show promise as a new treatment method [24]. In vitro and in vivo studies show that using AT1R inhibitors effectively and safely prevents and treats keloids and hypertrophic scars [25–28]. Human clinical trials have also shown positive results compared to placebo [25, 29–31].

Keloids and hypertrophic scars have a different skin structure than normal skin, with a thicker epidermis and dermis layer [32, 33]. Thus, topical medications are less effective [34, 35]. Scar tissue also has fewer hair follicles, adnexal glands, papillary dermis, and rete ridges than normal skin [36]. These conditions can hinder the penetration of drugs through the skin and lower water retention [37, 38]. Studies have shown inadequate hydration can further reduce drug absorption [34, 35].

To ensure a topical drug works, it must penetrate the skin and reach the target [39]. Thus, nano-sized carriers can help enhance delivery [40]. Lipid-based nanovesicles can effectively treat keloids with their specific skin structure [39], such as ethosome. These nanovesicles are known for their softness and elasticity [41]. Ethosome is superior to other vesicular systems. It can hold more drugs, penetrate deeper into the skin, and trap molecules of varying lipophilicity and is non-toxic for both in vivo and in vitro studies [42–46]. Ethosome is effective in occlusive and non-occlusive conditions and works well for dermal, transdermal, and intracellular delivery. They are also more stable and have smaller vesicle sizes [47]. Losartan potassium as an AT1R inhibitor loaded in ethosomal gel had demonstrated excellent entrapment ability, skin permeation ability, and formulation stability, enhancing the effectiveness of drug application. Animal safety assessments indicated it was irritant-free [48]. This study aims to assess the efficacy of 5% losartan loaded in ethosomal gel (5% LEG) compared to standard treatment based on clinical outcomes changes.

### Objectives {7}

The main goal is to compare the efficacy between 5% losartan loaded in ethosomal gel (5% LEG) and 10 mg/ml triamcinolone acetonide (10 mg/ml TCA) injection for keloid treatment, based on these parameters, i.e. The Patient and Observer Scar Assessment Scale 3.0 (POSAS 3.0) scores, erythema and pigmentation degree using dermoscopy (Heine® iC1) images, keloid surface area using digital photography planimetry (Sony® α6000), keloid thickness using high-frequency ultrasounds (Hitachi® Arietta s60 Japan), and pliability using durometer instrument type oo (Tecklock®Japan).

### Trial design {8}

Our study will follow a randomised controlled trial (RCT) design with a single-blind approach. We will use parallel group superiority trial and unpaired group methods and conclude the investigation by assessing parameter changes within 12 weeks. A block randomisation method is utilised to allocate participants, generating blocks of two with a 1:1 ratio. Randomisation does not distinguish the occurrence of keloids based on skin colour as the research setting tends to have a homogenous population with skin tones typical of Southeast Asians, ranging from light to dark brown [49].

## Methods: participants, interventions, and outcomes

### Study setting {9}

The study focused on patients aged 18 years and above, diagnosed with keloids, who visit the outpatient clinic of the dermatology department at The General Hospital of Syafira Pekanbaru, Riau, Indonesia, between August 2023 and April 2024.

### Eligibility criteria {10}

To qualify for our study, participants must be 18 years old or above and have keloid scars that measure 25 cm^2^ or less. Each participant must have at least one keloid that has lasted for at least 6 months, and they cannot take keloid medication for the past 2 months. Those who are unable to understand the patient and observer scar assessment scale (POSAS), refuse to participate, have nodular keloids, take antihypertensive medication, are pregnant, have a history of allergies, have active skin lesions in the keloid area, or have bleeding or scars are not eligible for the study. Additionally, we will exclude individuals who have undergone keloid treatment within the last month.

### Who will take informed consent? {26a}

The principal investigator will brief participants willing to participate in the study. The study will be explained through an explanatory sheet, and written informed consent will be obtained from participants before enrolling them.

### Additional consent provisions for collection and use of participant data and biological specimens {26b}

Not applicable. No involvement of any biological specimens.

## Interventions

### Explanation for the choice of comparators {6b}

The treatment comparator chosen is the active control intralesional injection of 10 mg/ml triamcinolone acetonide (10 mg/ml TAC). This is because it is the preferred method for treating keloids in some countries, such as Indonesia. However, intralesional steroid injections have several side effects, including pain and skin changes [19]; some people may be uncomfortable with needles [21].

### Intervention description {11a}

Forty-six eligible adolescents with keloid will be randomly assigned to the 5% losartan potassium loaded in ethosomal gel (5% LEG) group (*n* = 23) and active control of triamcinolone acetonide 10 mg/ml (TAC 10mg/ml) groups (*n* = 23). Ethosomal gel bearing 5% losartan potassium will be applied twice daily on the keloid with the rule of fingertips unit (FTU) [50], and 10 mg/ml TAC will be injected intralesionally in the keloid every 2 weeks for 12 weeks with the rule of 0.1 mg/cm^2^ [51]. With a Good Manufacturing Practise (GMP) certificate, the Dion Farma Abadi company will provide 5% LEG. The study patients will receive the intervention gel monthly during the study visit. Ten milligrams per millilitre triamcinolone acetonide (Flamicort®) used in the study is manufactured by Dexa Medica. Our trial has four study visits, i.e. before the intervention or baseline, after 4, 8, and 12 weeks.

### Criteria for discontinuing or modifying allocated interventions {11b}

If any signs of side effects, such as allergies or worsening complaints, are observed during intervention treatment, it will be discontinued immediately. Similarly, in the control injection treatment, the dose will be reduced or stopped if any allergic side effects or skin atrophy are detected or the participant expresses unwillingness to continue the study.

### Strategies to improve adherence to interventions {11c}

We will inform study patients how to use their gel treatment and the schedule of injections, and phone calls will follow them weekly. The compliance evaluation will depend on the quantity of gel left in the tubes and the adherence to injection visit schedules.

### Relevant concomitant care permitted or prohibited during the trial {11d}

While undergoing keloid treatment, patients can continue taking any previously prescribed drugs or supplements. However, it is advised to avoid adding any treatments related to keloid treatment, such as pain or itching relievers drugs, that may interfere with the efficacy assessment of the primary treatment.

### Provisions for post-trial care {30}

Any side effects during the study will be treated as they would in a clinical setting. The serious adverse event form will document any risks beyond minimal or physical injury. If a serious adverse event is found to be causally linked to the research intervention, it will be addressed according to applicable procedures. If the participants derive significant benefits from the new drug intervention, they can continue receiving the treatment as long as the gels are available. Similarly, suppose subjects benefit from the intralesional injection, the active control, and wish to continue with the therapy. In that case, it will be extended for approximately two months after the study concludes.

### Outcomes {12}

The research inquiry will encompass an assessment of both primary and secondary outcomes. The primary outcome directed towards the evaluation of POSAS 3.0 scores. Throughout the study’s duration, we will systematically assess POSAS 3.0 scores at four specific time intervals: the initial baseline assessment upon participants’ first arrival, followed by subsequent evaluations at weeks 4, 8, and 12. This measurement directly correlates with the patient’s quality of life [52]. We will utilise the most up-to-date version of the POSAS 3.0 questionnaire, accessed at https://www.posas.nl. We have received approval from the POSAS team to use this questionnaire and have been granted a license via email. This questionnaire will be translated into Indonesian and tested for validity and reliability before being used in the study. Patients will complete the POSAS 3.0 questionnaire, which includes 16 items. Five of these items are about the current state of their scar tissue, and 11 questions are about how their feeling related to the scar tissue over the past week. Meanwhile, an observer will complete a different section of the questionnaire with seven items scored on a scale of 1 to 5. We will add the PSAS and OSAS scores to determine the total score. The minimum patient score is 16, while the highest score is 80. The minimum and maximum scores for observers are 7 and 35, respectively.

In the context of this study, secondary outcomes encompass the evaluation of erythema and pigmentation levels, as well as assessments of keloid area, thickness, and pliability. These secondary outcome measures at four different time points will be assessed concurrently with the primary outcome.

The colour images will be captured using dermoscopy equipment from Heine® iC1 Germany, followed by image analysis. Dermoscopy is employed to capture colour images from three discrete regions, specifically focusing on the edges and central region. This process is iterated for precision, and subsequently, ImageJ image analysis computes the average colour quantification of the captured images. The same proficiently trained assistant will capture colour images using dermoscopy consistently during the entire duration of the study. Our team will utilise digital photography (Sony®α6000 Japan) and ImageJ image analysis software to examine the surface area. A trained assistant will take pictures of the keloid with appropriate camera lighting, and a ruler will be included in the shot to confirm the scale in ImageJ. The surface area changes will be measured in centimetres squared and evaluated at four different time points throughout the study.

A dedicated computer IT assistant, consistent throughout the study, will measure colour degrees and surface areas using ImageJ to maintain consistency.

High-frequency ultrasound (Hitachi® Arietta s60 Japan) will be employed to assess keloid thickness. The linear probe will be operating at frequency of 5–12 MHz [50]. Throughout the study, the same radiologist will consistently measure the thickness of the keloid in centimetres, specifically assessing it at the same three points as during the initial measurement. Subsequently, the obtained measurements will be averaged for analysis. The pliability of keloids is measured using a manual tonometer called durometer type oo (Tecklock® Japan). A trained assistant will use the tonometer on three different areas of the keloid and calculate the average in durometer units displayed on the device. During the study, we will also monitor for any potential side effects arising from the interventions.

### Participant timeline {13}

**Fig. 1:**
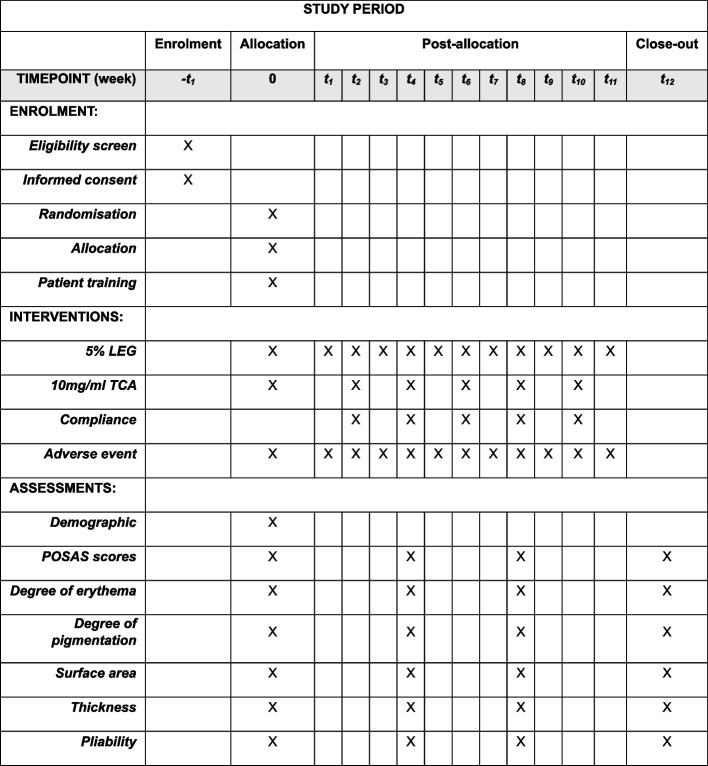
Schedule of enrolment, interventions, and assessments

The schedule of enrolment, intervention, assessment, and visits for participants is presented in Fig. 1, following the SPIRIT figure.

### Sample size {14}

The sample size determination depends on the specific criteria for comparative research. The variable type of the primary outcome data plays a significant role in deciding the appropriate sample size [50, 51, 53, 54]. The primary outcome is the changes in POSAS 3.0 scores, considered continuous data. Effect size measurement will be the mean difference. The research involved unpaired or two groups receiving different treatments, with the outcome being measured multiple times. As a result, a specific formula was utilised below [55].

This study maintains a 5% type I error dan is powered to have an 80% chance of detecting a minimum of 4 different scores of POSAS. Thus, a sample size of 18 was calculated for the two-sided approach. Adjusted sample size to anticipate a 20% dropout is obtained from the formula N1 = *n*/(1-d), with *n* as the sample size required per formula and d as the dropout rate [56]. Thus, the final sample will be 23 for each group treatment.

$${n}_1={n}_2=Y\left[2{\left(\frac{\left[{z}_{\alpha }+{z}_{\beta}\right]s}{x_1-{x}_2}\right)}^2\right]$$

*Z*_*α*_ = 5% (1.96), *Z*_*β*_ = 20% (0.84), *S* (standard deviation) = 4.8, *x*1-*x*2 (the minimum difference in POSAS scores between the two treatment groups) = 4, *Y* (correction factor for multiple assessments) = 0.8.

### Recruitment {15}

To determine the number of participants required based on the sample size, the investigator will document all new and existing patients who meet the inclusion criteria in the outpatient dermatology clinic in the general hospital Syafira and extend an invitation to participate. If the number of patients still needs to be improved, the researcher will seek assistance from other dermatologists to refer potential keloid patients willing to participate in the study. Additionally, the researcher will publicly announce a call for participation from individuals with visible scars that meet keloid criteria through advertisement.

## Assignment of interventions: allocation

### Sequence generation {16a}

To carry out the study, a non-research team member will use computer-generated blocks of two for randomisation, ensuring a fair allocation of participants with a 1:1 distribution across both groups. One group will have 23 participants receiving a 5% LEG, while the other will receive 10 mg/ml TAC and 23 participants.

### Concealment mechanism {16b}

The allocation of treatment is hidden from both participants and investigators. Upon enrolment, participants are notified by the investigator that they will be allocated to one of two treatment groups according to an allocation list generated by a computerised system. Each participant will have the same probability of receiving either treatment. This sequence list is adjusted to the participant’s arrival time for the baseline examination at the hospital. The nurse assistant will keep the record list and determine the treatment received based on the numbered sequence list.

### Implementation {16c}

After a non-research team member generates participant sequences from a computer programme, a nurse assistant as an administrator at the outpatient clinic will maintain their records. Each participant will be assigned a code number, and the code generated by the computer programme will determine the type of intervention they received. The nurse assistant will also have access to the patient’s identity and code number.

## Assignment of interventions: blinding

### Who will be blinded {17a}

The outcome assessors and data analyst will be blinded to the treatment assignment. The assessors will evaluate the clinical changes such as the POSAS scores and degree of erythema and pigmentation, as well as the area, thickness, and pliability of the keloid. As the intervention’s nature makes it impossible to blind participants and staff to the allocation, they are instructed not to disclose the participant’s allocation status during follow-up assessments. A computer IT assistant and other outcome assessors who assess the POSAS score and the degree of erythema and pigmentation, area, thickness, and pliability of keloid will anonymously be blinded to the intervention. They will record the results in anonymously case report forms (CRFs) and input data into separate datasheets online. This will enable the researcher to analyse data without accessing information about the allocation.

### Procedure for unblinding if needed {17b}

The outcome assessors are blinded to the interventions. Unblinding will be necessary should the assessors identify any severe adverse events, such as allergic or irritant reactions. The findings will be communicated to the investigators and The Medical and Health Research Ethics Committee (MHREC). The MHREC may permit unblinding if required. Nevertheless, unblinding is not necessary for minor adverse events, such as temporary pain, itchiness, or transient erythema. Unblinded participants who experience severe adverse events will not be excluded from the analysis, but they will be removed from ongoing treatment.

## Data collection and management

### Plans for assessment and collection of outcomes {18a}

For the primary outcome, the evaluation analyses change in POSAS 3.0 scores over four periods, from the initial baseline to the final assessment conducted by a trained and blinded dermatologist to the interventions. After each review, a nurse assistant will collect the completed POSAS 3.0 forms attached in a CRF, and another non-research team member will input data online into an online form directly to the data analyst. The patient and observer scar assessment scale (POSAS) is a practical, comprehensive, and reliable measurement tool involving the perspectives of patients and doctors [57–59].

There are several measurements for secondary outcomes. A proficiently trained assistant, blinded to the intervention, will utilise dermoscopy to capture the colouration of the keloid. Subsequently, these images will be anonymously forwarded to a computer IT assistant for assessment of the degree of erythema and pigmentation using the ImageJ image analysis programme. After each process, the results will be recorded in a CRF and sent via an online form without any identifiable intervention to the data analyst. Measuring colours using ImageJ is comparable to measuring colour with a colourimeter [60, 61]. Taking pictures using a dermoscopy is not affected by distance and lighting because the dermoscopy has a fixed shooting distance and polarised lighting that comes directly from the device itself, so the pictures taken are more accurate than those that use a camera. Dermoscopy is a valid and reliable method for assessing the colour of both pigmentation due to the presence of melanin pigment and erythema caused by increased vascularity of scar tissue [62].

A trained personnel will acquire a surface area photograph using a digital single-lens reflex (DSLR) camera and subsequently transmit it anonymously to the computer IT assistant. The IT assistant will then utilise the ImageJ image analysis tool to ascertain the measurement, yielding an average value in millimetre squares, which will be meticulously documented in a CRF. This information will be subsequently transmitted to an online form accessible solely by the data analyst, who will be granted access exclusively to the participant code while remaining uninformed about the intervention particulars. The change surface area with photographic planimetry is reliable for assessing an area of approximately or equal to 25 cm^2^ [63]. Pictures were taken using a versatile single-lens reflex camera that can capture better-quality images than other types of cameras [64]. In measuring the area of the wound, this device provides fast, accurate results and good reliability [65].

Keloid thickness in millimetres is assessed through high-frequency ultrasound (HFU) conducted by an intervention-blinded radiologist, utilising solely the participant’s code number in a designated CRF. Following this, the obtained data will be compiled by a nurse assistant, and the data entry process into an online form will be conducted by a non-research team member, who will subsequently transmit it to the data analyst for further analysis. The high-frequency ultrasound method is a non-invasive, affordable, and valuable method for evaluating the clinical efficacy of scar tissue treatment [66–69].

A trained assistant will measure the pliability of the keloid using a durometer type oo from three different areas. The result will be recorded in a CRF, and a non-research member will input the data into an online form to the data analyst without any known intervention. The tool will undergo routine calibration. Tissue tonometry is a simple, repeatable, and objective method of measuring tissue density and is clinically useful for assessing individual scar tissue [70–72].

### Plans to promote participant retention and complete follow-up {18b}

To ensure participants’ continued involvement, we will highlight the clinical advantages of participating in the study and provide transportation fees to alleviate any financial burden of attending examinations. Our research administrator will send patients reminders 2 days before their scheduled visit and reschedule if they cannot make it at the arranged time.

### Data management {19}

The computer IT assistant who does image analysis and a non-research team member will send data directly to data analysts online, ensuring anonymity and intervention blind. This will be done through a specific code entered into an online form spreadsheet. Any hard copies of the data, including CRFs, will be securely stored in a data bank at the hospital. The data analyst will double-check the completeness and clarity of the data by verifying that it matches the initial data in CRFs.

### Confidentiality {27}

Before signing the consent form, all potential and enrolled participants will receive an explanation sheet detailing the use of their personal information in this study. The explanation will emphasise the confidentiality of their identity, and the research staff will request their name and contact information solely for study purposes. However, their name will not be used in any published results. Instead, each participant will be assigned a unique code as a replacement identity. Any study results may be published in scientific literature, but the participants’ names or identifying information will be excluded. It should be noted that sponsors, the ethics committee of Universitas Gadjah Mada, or the researchers conducting the study may have access to the data obtained from the research. Nevertheless, the supervisor has the legal authority to protect all identifiable information from public disclosure unless required by law or a competent court. These notes will be kept confidential per the law and stored in the data bank of the general hospital of Syafira.

### Plans for collection, laboratory evaluation, and storage of biological specimens for genetic or molecular analysis in this trial/future use {33}

Not applicable. No involvement of any biological specimens.

## Statistical methods

### Statistical methods for primary and secondary outcomes {20a}

Statistical analysis will be done by Statistical Package for Social Science (SPSS) version 24. An analysis of the demographic data of the research subjects is planned to be conducted using descriptive statistics. The findings presentation will utilise frequency and percentage tables. Mean values are presented with standard deviation for continuous variables with a normal distribution. The normality of the data can be determined using the Shapiro-Wilk test. If the distribution is not normal, the median and interquartile range (IQR) are used. We conduct an analysis that compares the efficacy of a numerical variable in two groups and within a group, measured multiple times, using a generalised linear model analysis with alternative repeated Mann-Whitney analysis. Our study focuses on continuous variables for both primary and secondary outcomes. Our test had a power value of 80%, a confidence interval of 95% (95% CI), and a significance level of *p* < 0.05.

### Interim analyses {21b}

Not applicable. An interim analysis is unnecessary due to established product safety, a small number of participants, and a short study period.

### Methods for additional analyses (e.g. subgroup analyses) {20b}

Additionally, we evaluated the level of agreement between patients and observers on scar tissue assessments using POSAS 3.0 by calculating the Pearson correlation coefficient. When analysing the impact of keloid duration on treatment outcomes, Spearman’s correlation coefficient is utilised with a significance level of *p* < 0.05.

### Methods in analysis to handle protocol non-adherence and any statistical methods to handle missing data {20c}

Our analysis will involve intention-to-treat analysis (ITT) to address non-adherence among participants, along with multiple imputations to handle any missing data. Additionally, we will thoroughly report the reasons for withdrawal within each randomisation group and qualitatively compare them. To ensure accuracy, we will combine all completed and imputed datasets and utilise Rubin’s multiple imputation method with five datasets to estimate the treatment effect.

### Plans to give access to the full protocol, participant-level data, and statistical code {31c}

Data derived from this investigation will be provided to proficient scholars with an academic concern in keloid treatment. We plan to submit dataset in the National Library of Medicine repository. Any shared data or samples will be coded, and no protected health information will be included. Authorization of the dataset solicitation is mandatory prior to sharing any data with the inquiring entity. The data will be made available for up to 24 months, commencing 2 years after the publication of the results. Requests for extensions will be evaluated on a case-by-case basis. Trial individual participant data (IPD) can be requested by eligible researchers who are conducting independent scientific research.

## Oversight and monitoring

### Composition of the coordinating centre and trial steering committee {5d}

The study’s principal investigator and research physician will collaborate in developing and executing the research plan, including protocol preparation and revisions. Additionally, they will provide an investigator brochure (IB), case report forms (CRFs), and informed consent forms. The assessment assessors will receive training from the principal investigator and other research physicians, who will also disseminate the study results. The trial management committee, composed of the principal investigator, research physicians, data manager, and administrator, will oversee the day-to-day running of the trial, including study planning, risk reporting to the ethics committee, randomisation, and data verification. The data manager will maintain the trial IT system, data entry, verification, and statistical analysis. The committee will meet at least three times: before enrolment, during the study, and at the end to determine the necessary steps in the trial.

The trial steering committee is an independent group comprising a clinical consultant, medical statistician, clinical monitor, regulatory authority, and patient representative. Their role is to ensure that the study runs smoothly and efficiently by reviewing the final protocol and any necessary changes during the study. The committee monitors the trial’s progress, adherence to the protocol, and patient safety, ensuring ethical requirements, regulations, and standards are followed. They also provide that the necessary documentation is available and that all collected data is accurately recorded. The committee holds meetings prior to study enrolment and during the middle of the study.

### Composition of the data monitoring committee, its role and reporting structure {21a}

Not applicable. Based on the literature review conducted by Sydesand et al. [73], it has been determined that the data monitoring committee (DMC) is unnecessary for this trial since the study is of short duration (12 weeks) and presents minimal risk to participants who enrol. Throughout the study, the sponsor-investigator will ensure that all planned activities are carried out correctly and that any shortcomings are quickly identified and fixed. Thus, a clinical monitor from the education and research unit of Syafira Hospital is accountable for supervising the study’s progress and protecting patients’ safety and rights. The clinical monitor ensures that ethical requirements, regulations, and standards are followed, essential documentation is accessible, and all gathered data is recorded accurately.

### Adverse event reporting and harms {22}

According to a recent study, the new therapy intervention has been deemed safe and does not cause any irritations [49]. Yet, there may be other unknown side effects that could arise during the study. The intralesional injection of triamcinolone acetonide may lead to temporary side effects, including pain, inflammation during injection, bleeding, contact dermatitis caused by preservatives in the injection fluid, infection (rare), and anaphylaxis reactions (infrequent). Long-term side effects may include skin atrophy, telangiectasia, hypo- and hyperpigmentation, and steroid acne (rare) [20]. Participants need to report any adverse events during the study, and researchers need to document them in an adverse event form. Any adverse events will be handled appropriately, following clinical procedures, and reported to the trial steering committee and Medical and Health Research Ethics Committee (MHREC) at Universitas Gadjah Mada.

### Frequency and plans for auditing trial conduct {23}

The Indonesian Food and Drug Authority will oversee the auditing of trial conduct as a regulatory authority and will be included in the steering committee. This auditing process will be independent of the investigator and sponsor, and it will take place in the early stages and in the middle of the trial to ensure that subjects are protected and that regulations, including the principles of Good Clinical Practices (GCPs), are being followed.

### Plans for communicating important protocol amendments to relevant parties (e.g. trial participants, ethical committees) {25}

If any changes to the study protocol could impact the safety and well-being of participants, we will ensure that all relevant parties are informed. This includes the ethics committee, trial registry, steering committee, journal, and participants. Before conducting the trial, any modifications that concern patients’ safety and well-being will require confirmation from the MHERC at the Faculty of Medicine, Public Health, and Nursing at Universitas Gadjah Mada Yogyakarta and the steering committee. Any modifications will be reported to ClinicalTrials.gov as a registry board, and participants will be notified if there are any changes to interventions and outcome assessments. Finally, the Trials journal will serve as the publication site for the modifications.

### Dissemination plans {31a}

The research results will be shared with the public through journal publications. At the same time, research reports will be submitted to the funder Universitas Gadjah Mada. Furthermore, the research findings will be reported to the institution in charge of clinical registration through Clinicaltrial.gov. This marks the final step of the registration process.

## Discussion

Based on the previous review [14], it has been found that angiotensin II can initiate the formation of keloids and hypertrophic tissue through various mechanisms. It can lead to vascular dysfunction due to the effects of vascular endothelial growth factor (VEGF) and basic fibroblast growth factor (bFGF) II, which can both promote angiogenesis. However, VEGF can also contribute to fibrosis development through various means, such as activating signals that stimulate growth, movement, and collagen production by myofibroblasts through TGF-β1 signals and extracellular signal-regulated kinase (ERK) [72]. Another mechanism is increased IL-6, which plays a role in the process of fibrosis, strengthens the deposition of extracellular matrix, type I collagen, and stimulates other fibrogenic mediators, including TGF-β and tissue inhibitor matrix metalloproteinase (TIMP) [74]. Furthermore, angiotensin II may increase TGF-β and connective tissue growth factor (CTGF) expression. TGF-β has an essential role in the stimulation and synthesis of the extracellular matrix. It causes the formation of scar tissue through the TGF-β signalling pathway. Angiotensin II through the AT1 receptor will activate collagen expression through the TGF-β/small mothers against decapentaplegic (SMADs) signalling pathway [73, 75]. Stimulation of keloid fibroblasts, hypertrophic scar tissue, and normal tissue by TGF-β will cause an increase in the expression of CTGF, which becomes a fibrogenic mediator on angiotensin II [76]. Ultimately, the mechanism involved is increasing the expression of tissue inhibitors of matrix metalloproteinases-1 (TIMP-1), which functions to degrade, inhibiting the work of matrix metalloproteinases (MMPs) [75]. Angiotensin II binds to AT1R in melanocytes and will increase melanogenesis through the protein kinase C (PKC) pathway [77].

Losartan potassium is among the initial types of angiotensin II type 2 receptor blockers (ARBs) that have undergone extensive research regarding fibrosis. Losartan falls under the ARB drug class, which shares similar effects with other drugs of the same class but has a distinct molecular effect that sets it apart from others. The molecular effects of losartan in the process of fibrosis are that it can reduce the production of chymase, which plays a role in the formation of angiotensin II as in mice with cardiac fibrosis [78] and can modulate prostaglandins which play a role in the process of fibrosis [79]. Losartan potassium inhibits prostaglandin thromboxane A2/prostaglandin endoperoxide H2 (TxA2/PGH2) receptors on blood vessels and platelets which plays a role in the process of fibrosis by reducing the production of cyclic adenosine monophosphate (cAMP), which acts as an anti-fibrosis through the inhibition of SMADs [79] so that the process of fibrosis increases. Losartan also can increase the production of prostaglandin E2 (PGE2), which has an anti-fibrotic effect, and PGE2 keloids have decreased production by fibroblasts [80–82]. Research on losartan administration to rats with pulmonary fibrosis showed increased PGE2 production [83]. Losartan potassium can be found on the market at an affordable price compared to other ARB classes.

One of the advantages of administering topical drugs is that they are more comfortable in application because they do not cause side effects of pain, have high adherence, and can be applied by the patient himself [23]. Problems in topical drug delivery that must be addressed so that drugs can work optimally are the physicochemical characteristics of drugs and physiological barriers on the skin [84] as well as drug formulations that can affect penetration ability [35]. Drug formulations can affect penetration ability, so the delivery strengthening strategy is chosen through passive methods using nano-sized carrier formulations [40] using an ethosome suitable for keloid skin structures [39]. The choice of this gel-based drug carrier is due to its minimal effect on the stability of the lipid nanovesicles. It can improve the permeation rate of the drug in the skin [85], apart from convenience in the application. Determination of the percentage of 5% losartan potassium selected in the preparation was based on previous studies [29, 31]. Based on the suspected mechanism of action in previous studies, we hypothesised that 5% LEG has good efficacy for keloid treatment and is comfortable to use.

This study has several strengths, including a randomised, unpaired group, single-blind, and active-control design. Furthermore, the effects of 5% LEG are examined from both subjective and objective perspectives. Assessing outcomes in a clinical environment with limited resources has proven practical and efficient with the available evaluation tools. While the research conducted has its strengths, there are also some weaknesses. One such area for improvement is that the study does not include a comparator of 10 mg/ml TAC and 5% LEG combination therapy group. Additionally, the study only measures clinical outcomes and does not confirm any evidence from biomarker changes. Furthermore, the study is limited in duration, and it will be beneficial to extend the application of 5% LEG to observe its impact on keloids over a more extended period. This trial will provide scientific evidence favouring using 5% LEG for managing keloid.

## Trial status

The protocol version number is CT202300, 11 January 2023. Recruitment started on 30 March 2024 and is expected to be completed by 30 September 2024.

## Data Availability

The sponsor, the researchers, and the ethics committee of Universitas Gadjah Mada may have access to the final trial dataset obtained from the study.

## Abbreviations

IL: Interleukin
TGF-β: Transforming growth factor-β
RAS: Renin angiotensin system
AT1R: Angiotensin II receptor type 1
AT2R: Angiotensin II receptor type 2
TAC: Triamcinolone acetonide
LEG: Losartan potassium loaded in ethosomal gel
POSAS: The Patient and Observer Scar Assessment Scale
GMP: Good manufacturing practise
FTU: Fingertips unit
MHREC: Medical and Health Research Ethics Committee
HFUS: High-frequency ultrasounds
CRFs: Case report forms
ITT: Intention-to-treat
IB: Investigator brochure
DMC: Data monitoring committee
GCPs: Good clinical practises
VEGF: Vascular endothelial growth factor
bFGF: Basic fibroblast growth factor
ERK: Extracellular signal-regulated kinase
TIMP: Tissue inhibitors of matrix metalloproteinases
CTGF: Connective tissue growth factor
SMADs: Small mothers against decapentaplegic
MMPs: Matrix metalloproteinases
PKC: Protein kinase C
ARB: Angiotensin II receptor blocker
cAMP: Cyclic adenosine monophosphate
TxA2: Thromboxane A2
PGH2: Prostaglandin endoperoxide H2
PGE2: Prostaglandin E2
